# “That was our afterparty”: a qualitative study of mobile, venue-based PrEP for MSM

**DOI:** 10.1186/s12913-023-09475-8

**Published:** 2023-05-17

**Authors:** Grace Chamberlin, Mairead Day Lopes, Surabhi Iyer, Christina Psaros, Ingrid V. Bassett, Susana Medeiros, Catherine O’Connor, Kevin L. Ard

**Affiliations:** 1 Medical Practice Evaluation Center, Massachusetts General Hospital, Boston, MA USA; 2 Health Innovations, Inc, Boston, MA USA; 3 Department of Psychiatry, Massachusetts General Hospital, Boston, MA USA; 4 Harvard Medical School, Boston, MA USA; 5 Division of Infectious Diseases, Department of Medicine, Massachusetts General Hospital, Boston, MA USA

**Keywords:** PrEP, Venue-based care, Mobile unit, MSM, Sexual healthcare utilization

## Abstract

**Background:**

HIV preexposure prophylaxis (PrEP) uptake among men who have sex with men (MSM), a group disproportionately impacted by HIV, is not commensurate with need. Settings which reduce or remove barriers to accessing care are promising venues to support PrEP uptake. PrEP provision at mobile clinics represents a novel strategy to increase PrEP access; however, the acceptability and feasibility of this approach have not been well studied.

**Methods:**

Our objective was to understand patient and staff experiences of a mobile clinic van offering PrEP and sexual health services in Boston, Massachusetts, USA. We interviewed mobile unit users and conducted focus groups with mobile unit staff and users. Data were organized using Dedoose software, and content analysis was used to identify themes of access, community, and stigma.

**Results:**

Nineteen individuals (16 patients and 3 staff members) participated in interviews (N = 13) or focus groups (N = 6). All patients identified as MSM, 63% were Hispanic or Latino, and 21% of patient interviews were conducted in Spanish. Logistical and psychological convenience facilitated service use, while the community-oriented environment improved satisfaction with care. Overall, participants supported expansion of mobile unit services and recommended changes to improve access to longitudinal care. However, some barriers to PrEP persisted, including low HIV risk perception and stigma about sexual behavior.

**Conclusions:**

Mobile units can promote sexual health and PrEP uptake, particularly for populations facing social and logistical barriers to care in traditional settings.

**Supplementary Information:**

The online version contains supplementary material available at 10.1186/s12913-023-09475-8.

## Introduction

Men who have sex with men (MSM) are disproportionately affected by HIV, with 70% of new infections in the United States in 2019 occurring among gay, bisexual, and other MSM [1]. PrEP is highly effective for preventing HIV, and while a majority of MSM are aware of this prevention measure, PrEP uptake is not commensurate with need [2, 3].

Barriers to PrEP for MSM include limited knowledge of how to access services, low perception of risk for HIV acquisition, and lack of geographical access, with 1 in 8 PrEP-eligible MSM in the USA living in “PrEP deserts” [4–8]. MSM, particularly young racial and ethnic minority MSM, may also face barriers to preventive services due to stigma around their intersectional identities and logistical issues with clinic hours and location [9–11]. While the impact of identity-based stigma and poor geographical access on PrEP uptake may be reduced in urban areas, low perception of risk and lack of knowledge about options still serve as barriers in cities [7, 12].

As a result, the implementation and assessment of methods to mitigate barriers to care are critical. The USA’s Ending the HIV Epidemic (EHE) initiative has set a goal to reduce new HIV infections in the US by 90% by 2030 [13]. Consistent with EHE strategies to expand PrEP provision and optimize linkage to care, Health Innovations – a community-based organization offering sexually transmitted (STI) testing on a clinic van – and the Massachusetts General Hospital Sexual Health Clinic began collaborating in 2018 to provide same-day oral PrEP initiation and STI treatment on the van during nighttime and evening hours [14]. Services were offered outside of gay bars and clubs in Suffolk County, Massachusetts, which includes the urban core of metropolitan Boston and is a priority jurisdiction in the EHE plan due to its burden of HIV [15].

Few studies have focused on the use of mobile units for sexual health services. One 2019 study in Australia found that 78% of patients thought mobile clinic vans were a good venue for STI testing and would be happy to refer others [16]. Studies from 2014 to 2015 in Peru and Australia, respectively, found that mobile units reached more untested individuals, with 48% of mobile unit patients in the Peruvian study having never previously tested for STIs [17, 18]. For HIV-specific care, community-based, mobile antiretroviral treatment has been shown to increase virologic suppression in sub-Saharan Africa [19]. However, PrEP provision on a mobile unit has been less well described. One study of adolescent and young women seeking care at a mobile clinic for sexual and reproductive health services, including PrEP, in South Africa found that mobile services were acceptable to this population, but it is not clear if these findings can be generalized to other settings (e.g., the USA) or populations (e.g., MSM) [20].

Mobile units may represent an opportunity to improve access to sexual health services and increase uptake of PrEP, particularly for populations with high risk and lower service utilization, such as young, racial and ethnic minority MSM. However, implementation of mobile units offering sexual health services is limited, and evaluation of the acceptability and the feasibility of this model for longitudinal care and PrEP provision is lacking in the literature. Therefore, we conducted this qualitative analysis of a mobile PrEP program in Suffolk County, Massachusetts to identify strategies for improvement and provide insights into ways mobile clinics can help meet EHE goals.

## Methods

### Study setting

The mobile unit clinic was staffed by nurses, who provided HIV and STI testing (gonorrhea, chlamydia, and syphilis), counseling, and care navigation (insurance or PrEP drug assistance program enrollment), and a nurse practitioner, who evaluated symptoms, administered empiric STI treatment for patients with STI syndromes, and prescribed daily oral PrEP, with phone backup from a physician. At least one clinician at each clinic spoke Spanish fluently. Clinics were held on weekend nights (~ 10 PM to 4 AM) outside of bars and clubs, or occasionally during the day at community events (e.g., Pride celebrations). Patients who were intoxicated or otherwise did not have capacity to engage in care were not offered STI/HIV testing or PrEP but were instead offered follow up with the mobile unit or the Sexual Health Clinic at a later time. Care was provided at no cost to patients.

### Study participants

**Fig. 1:**
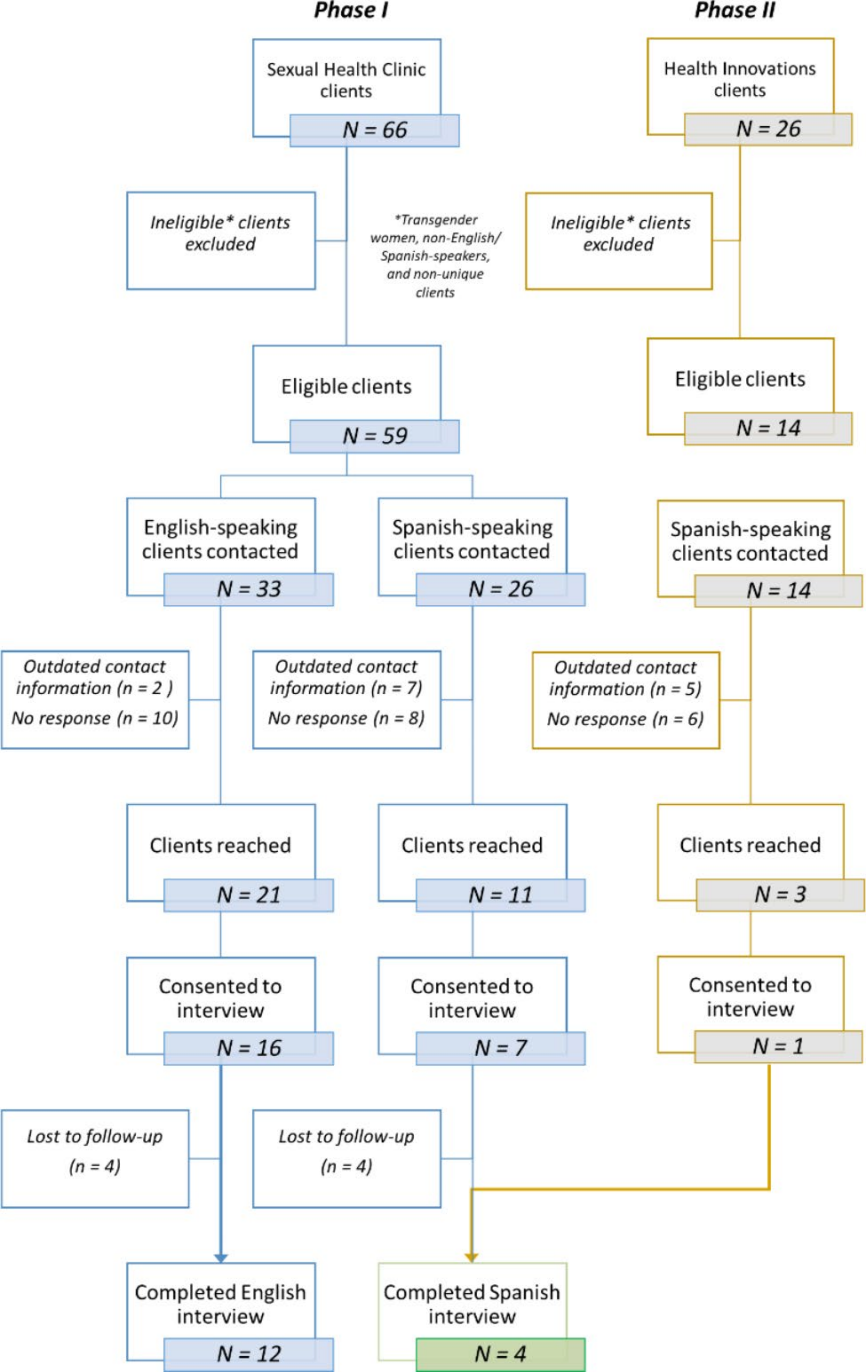
Enrollment Summary, with Phase 1 (blue) representing the first stage of participant recruitment based on initial patient lists from the Sexual Health Clinic, and Phase 2 (yellow) representing a secondary stage of recruitment based on a list of Spanish-speaking patients of the mobile unit. Phase 1 includes those who accessed the van at a time when PrEP was offered, while Phase 2 included a supplementary group of patients who still utilized the mobile unit but may have accessed only STI testing

Methods are presented in accordance with COREQ guidelines [21]. (See additional file 1 for a detailed summary of this study in the context of the COREQ guidelines.) We conducted individual, semi-structured interviews (N = 16) with mobile unit patients as well as two focus groups, one with patients (N = 3) and one with staff (N = 3). We initially intended to recruit a larger sample directly from the mobile unit and gather data solely through focus groups, but the Covid-19 pandemic paused the mobile unit’s sexual health services and forced a pivot to remote, individual interviews. Participants were recruited from a convenience sample of mobile unit users who identified as MSM and used the unit’s services between January 2019 and February 2020; the population was limited to those 18 years or older because PrEP was not available to minors on the mobile unit during the study period. Two female research assistants (GC and MDL) trained in qualitative methods contacted eligible patients to explain the goals of the research and assess their interest in participating; they had no prior relationship to the participants. All eligible participants (N = 73) were contacted at least twice; Fig. 1 provides a detailed summary of the enrollment process. Of 73 eligible patients, 35 (48%) were reached, and 16 (22%) completed interviews or focus groups. Interviews were conducted in English or Spanish, depending on the participant’s preferred language, while the participant was in a private setting. Study procedures were approved by the Partners (Massachusetts General Hospital/Brigham and Women’s Hospital) Institutional Review Board (Protocol 2019-P003021, Boston, MA).

### Measures

**Fig. 2:**
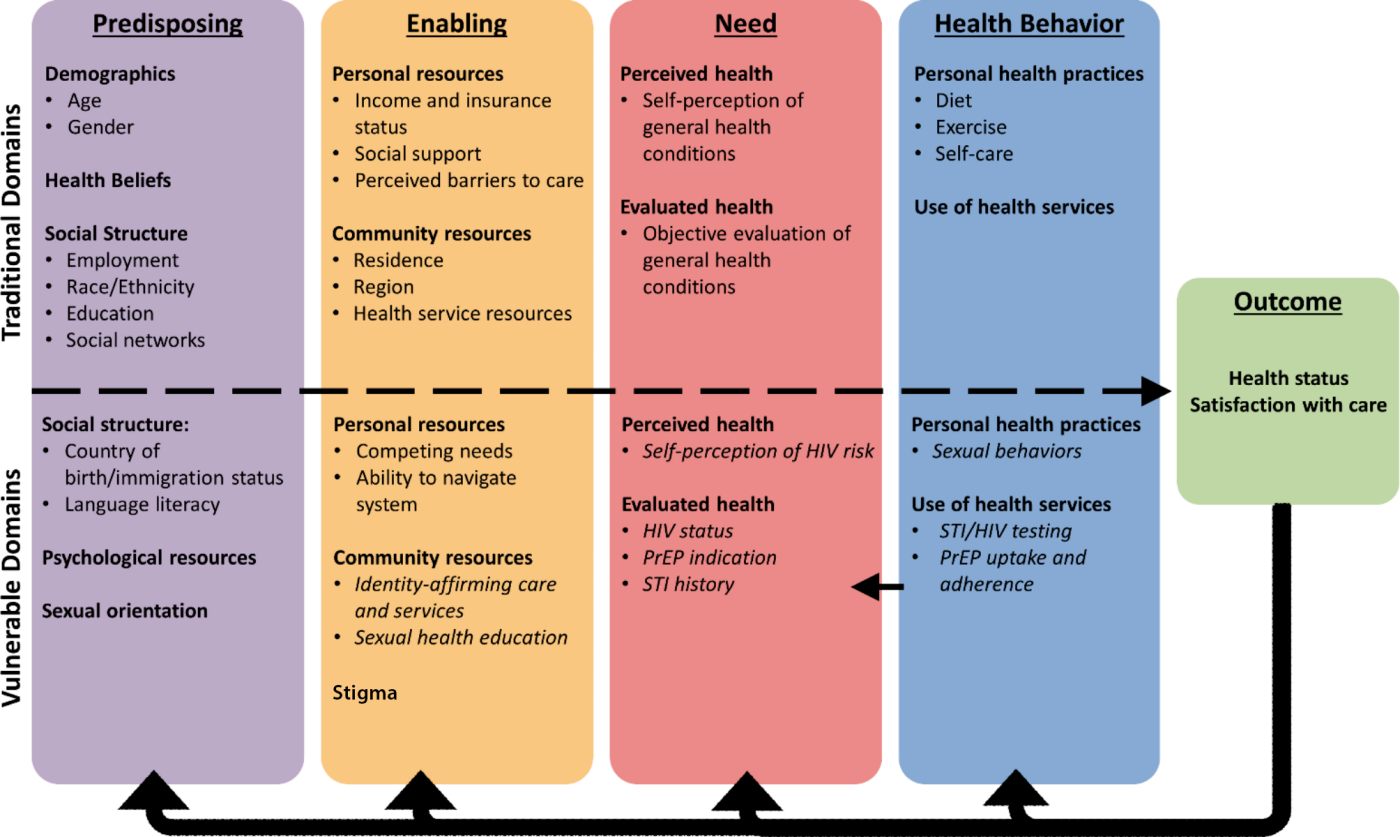
Modified Gelberg-Andersen Behavioral Model for Vulnerable Populations, as applied to MSM and their utilization of sexual health services. Each box represents one of the categories impacting health service utilization and health: outcomes (green), predisposing (purple), enabling (yellow), need (red), and health behavior (blue). Each box includes bolded headings showing factors which fall under each category, and bulleted examples falling under each heading. Traditional factors affecting the whole population’s service utilization are above the dashed line, while factors relevant to vulnerable populations are below the dashed line. Specific examples of these domains reflected in the results from our data have been added to the model in italics. The arrows linking the boxes represent the cyclical nature of the model, with predisposing and enabling factors, need, and health behaviors both impacting and being impacted by health outcomes. Our data showed a bidirectional relationship between health behaviors and need, represented by the arrow from the health behavior (blue) to the need (red) box

We developed semi-structured interview and focus group guides informed by a review of recent literature on barriers to PrEP uptake and the use of community-based models for health services, investigators’ a priori knowledge from providing PrEP and sexual health services, and domains derived from the Gelberg-Andersen Behavioral Model for Vulnerable Populations, [22, 23] (Fig. 2), such as perceived barriers to care, community resources, health behaviors and beliefs, and ability to navigate systems and use health services. Questions were open-ended and focused on barriers to accessing sexual health services, experiences of the mobile unit, and attitudes towards PrEP (sample interview questions shown in Table 1). Both mobile unit patients and staff were asked for suggestions on how to improve or expand van services. In addition to qualitative responses, we collected information about age, race, ethnicity, insurance status, education, and PrEP use from mobile unit patients (Table 2). Focus group participants are not identified by this information in the manuscript to preserve their privacy. Participants received 20 US dollars for their time. Patient interviews lasted 30–60 min; focus group discussions lasted 1–2 h.

**Table 1:** Study content areas and example questions

Content Area	Questions and probes
Warm-up questions	
	What do you think are the major HIV services that men who have sex with men in the Boston area need?
Experience of the Mobile Clinic Van	
	Do you have any experience with accessing sexual healthcare on a mobile healthcare unit/van? If yes, please describe your experience(s).
	How does providing sexual health services on a van help overcome challenges in accessing care? What challenges remain?
Accessing Sexual Health Services	
	What are your preferences for accessing services for sexual healthcare and HIV prevention? For example, do you prefer specific locations, clinic types, clinician types? Please explain why.
	What barriers do you experience when accessing services for sexual healthcare?
Attitudes towards PrEP	
	What do you think about using PrEP for HIV prevention? Why do you feel this way? Is PrEP something you would consider using? Why or why not?
	Have you ever tried to access PrEP? How did that go? For those who didn’t start, what happened? How did you hear about it?

### Analysis

Interviews and focus groups were audio-recorded, transcribed, and translated when needed from Spanish to English. Transcriptions and translations were performed by a professional transcription/translation service (TranscribeMe), approved by the institutional review board. Using a mixed inductive and deductive approach consistent with reflexive analysis, we generated a codebook based on recurring categories from all transcripts; this codebook was then imported into Dedoose software (version 9.0.46) [24]. Two coders (GC and SI) double-coded three (> 10%) of the transcripts and completed code application training tests in Dedoose to ensure inter-rater reliability. Categories and subcategories outlined in the codebook were continually reexamined to check for applicability and consistency in codebook interpretation and the remaining transcripts were then independently coded. Key themes were derived from the data and are presented below through the lens of related domains in the Gelberg-Andersen Behavioral Model for Vulnerable Populations [23, 25, 26]. This model describes health service use as a function of predisposing, enabling, and need factors which affect health behaviors and ultimately health outcomes, specifically for those who face additional barriers. Therefore, it serves as a useful framework through which to analyze our data on the experiences of MSM accessing sexual health services, as this group faces stigma, high rates of STIs and HIV, and barriers to care specific to their sexual orientation. Figure 2 highlights findings organized according to domains of the Gelberg-Anderson model, specifically enabling, need, and health behavior factors. Oversight of the qualitative process was provided by CP; topic-related content was reviewed by KLA, IVB, and CP.

## Results

Mobile unit patient participants had a mean age of 28 years (range 21–52 years), were 63% Hispanic or Latino (with 21% of participants completing the interview in Spanish), and the majority had used PrEP (current: 26%, previous: 47%); all identified as MSM, and none identified as transgender or gender diverse (Table 2). Saturation around themes of enabling, need, and health behavior factors was achieved.

**Table 2:** Demographics of mobile unit patients (N = 19)

Age in years		
	Mean (SD)	28 (± 6.8)
Race		
	White	7 (37%)
	Other	6 (32%)
	Multiple races/mixed race	5 (26%)
	American Indian/Alaskan Native	1 (5%)
Ethnicity		
	Hispanic or Latino	12 (63%)
	Not Hispanic or Latino	7 (37%)
Language of interview		
	English	15 (79%)
	Spanish	4 (21%)
Insurance status		
	Insured	18 (95%)
	Uninsured	1 (5%)
Highest level of education completed		
	Bachelor’s degree	9 (47%)
	Associate’s degree	3 (16%)
	Some college	5 (26%)
	High school	2 (11%)
PrEP use		
	Current	5 (26%)
	Previous	9 (47%)
	Never	5 (26%)
PrEP prescription source for previous and current users (N = 14)		
	Traditional clinic	7 (50%)
	Mobile unit	7 (50%)

### Enabling factors: access and community

Enabling factors affect the ability to manage competing needs – such as fulfilling work or social responsibilities – while engaging in health care. Enabling factors can either facilitate or impede service utilization. By positively influencing the domains of competing needs and system navigation, mobile units improve sexual health service utilization and overcome barriers to care. We identified two overarching themes reflecting how the mobile unit facilitated engagement with sexual health services for MSM: logistical and psychological access and a sense of community.

Due to its non-traditional location and hours, the mobile unit provides care when and where it is convenient. Most study participants noted this convenience as a key feature that improved access. Specifically, not having to schedule an appointment, travel to a visit, pay for care, or miss work allows patients to engage without the usual burden of competing needs. As one patient focus group participant said, *“If you want to take care of yourself, just take one hour, and at 3am. I think you’re able to take just one hour for your health. So, it’s pretty easy, in my opinion, with the van. Maybe you work 8 to 5, you don’t have a lot of time in the day, so why not at 3 in the morning?”*

Importantly, several participants spoke to spontaneity as a key driver for their decision to seek care on the mobile unit: *“I think we were all just pleasantly surprised with this opportunity to check in on our sexual health, how accessible that opportunity was. And it was just kind of a spontaneous on-a-whim decision,”* recalled a 23-year-old, White patient who has never used PrEP. This “why not” mentality was echoed repeatedly, suggesting that the van’s unexpected location and hours reduce not only logistical barriers to care, but psychological ones as well. As one 28-year-old, Hispanic/Latino patient who has never used PrEP summarized,

*“I’m sure there’s people out there, kids or young adults that were probably going through the same thing that I went through [not being comfortable discussing sexual orientation in the past]. So I think they might be either afraid or maybe just not as comfortable going to an actual clinic. So when you have that option just right there, it’s obviously a lot more convenient, and it puts less pressure on you, and it’s just like go in and do it, and it becomes more accessible. And it, I’d say, reaches more people too.”*

Others echoed this sentiment of psychological convenience; the low-threshold nature of the mobile unit eliminates the need for planning, thus overcoming low self-efficacy by minimizing the need for system navigation:

*I think those van concepts are really, really powerful, at least for me. For someone that’s not apt to necessarily contact someone for services, it provided a really easy way for me just to simply walk in and say, ‘Listen. I’m going to do something about this now. I’m finally here. Here’s the moment.’*

(30-year-old, White, previous PrEP user)

I guess, for me, personally, and for people who are maybe either scared or shy or just don’t know what to do but don’t want to put the effort into figuring out how to get access to sexual healthcare, that [the mobile unit] really just bridged the gap. And it was presented to me in such an easy, no-effort kind of way. It made me less scared of PrEP and HIV, and I learned more about it. …It worked because, before, I just didn’t know how to access things, or I was a little too scared

(23-year-old, White, never used PrEP)

The mobile unit also mitigates potential competing needs of MSM by allowing men to care for their sexual health in a social setting. The spontaneous engagement cited above was often facilitated by peers, an observation relayed by patients and mobile unit staff.

“*TI was just curious about it at first. And yeah, the fact that a service like that was actually out there. And again, my friends were there, and they were like, “Yeah, let’s all do this together,“ and I was like, “Sure. Why not?“*

(33-year-old, Hispanic/Latino, never used PrEP)

*I went to the van with my two roommates and that was our afterparty. If you don’t have an afterparty, you go take care of your health and do something for yourself.*

(Focus group participant, Hispanic/Latino, current PrEP user)

*I think that when we go to a club, for example, people are there with their friends. And we find that a lot of patients do come, at least with one other person, even if the friend or group of friends aren’t getting tested. It does seem like that group effect of being encouraged.*

(Staff focus group participant)

While the accessibility of the unit increases engagement for those who might otherwise struggle to prioritize care or navigate the system, community may be critical for satisfaction and continued engagement with care. Participants highlighted the non-traditional, community atmosphere of the mobile unit as a strength. A frequently cited aspect of the setting was the judgement-free environment and knowledgeable staff, which differed from experiences in traditional clinics, where participants anticipated or had experienced stigma.

*Community is a very important thing to queer people, so I feel like an established institution is kind of…they can be a bit…they can be a bit out of touch with us. It just… can be awkward to talk to your primary care doctor about that if they’ve never asked you. ... I could tell all those nurses and doctors were very knowledgeable of queer life, which was important to me and probably most queer young people. If they’re queer themselves or if they’re just educated about it, I think that’s very important.*

(23-year-old, White, never used PrEP)

The identities and MSM-specific knowledge of the staff allowed patients to feel they were surrounded by members of a supportive community: “*It felt like family, and they were very informative about PrEP. It didn’t feel too serious, and they made it clear that the safety of the queer community was very important to them,”* said a 23-year-old, White participant who never used PrEP. The mobile unit staff cultivated a welcoming and informal environment, facilitating initial patient engagement and satisfaction with care. One participant (24-year-old, White, previous PrEP user) noted that the nurse practitioner they spoke to was “*really nice, easygoing, just asking tons of sexual-related questions that normally people are uncomfortable to answer…[making] the whole process really easy*,” and a focus group participant who identified as Hispanic/Latino and a current PrEP user contrasted this openness with traditional clinic experiences, saying *“I feel like the doctor judges you in too many ways, and in the van I just feel like everyone was really friendly and helpful*.”

Suggestions for improving the unit fall under the same themes of access and community; further enhancements could promote retention in longitudinal care and improve health status. For example, one common recommendation was to increase the presence and availability of the van. Mobile unit staff agreed that their inconsistent schedule, while allowing spontaneous access to sexual health services, could also be a barrier to longitudinal care. Along with a consistent schedule, some staff members called for an appointment option.

“*That’s [an appointment option] not where we’re going to get new patients, but maybe as a way to better serve our existing patients: people who would like to see us again because they had a great experience the first time, they had their needs met, they felt the space was comfortable and non-judgmental. I think some way to access us online where we could come to them might also be helpful in retaining those folks.”*

(Staff focus group participant)

### Need

Despite enabling care through access and community, barriers to care among the participants still existed. Within the Gelberg-Anderson model, the barriers in our study fell primarily within the category of need. The most consistent barrier was perceived health. Despite positive views of PrEP and agreement that PrEP awareness and access are critical for MSM, many in our study population did not feel they needed PrEP even though they had utilized other sexual health services, such as STI testing, on the mobile unit:

*I think [PrEP] is the best thing to do for HIV prevention… I still haven’t gone on PrEP because I don’t think I, in a weird way, need it yet. I sound like such a contradiction because I’m praising PrEP so much but still—in the back of my mind—not ready to go on it*.

(23-year-old, White, never used PrEP)

This perception was reported even by patients to whom a clinician had recommended PrEP. One participant (33-year-old, Hispanic/Latino, never used PrEP) had a potential HIV exposure and was told by his provider, “‘*Try to find a way, if you can, to take PrEP. If you had taken PrEP, you wouldn’t be mixed up in this.’ And it’s true, and I told him, ‘Okay, let me think about it.’ But, nevertheless, I didn’t want to take PrEP… I’m almost never sexually active.”* Another participant (24-year-old, White, previous PrEP user) was similarly reluctant to start PrEP: *“My PCP [primary care provider] wanted me to take it earlier on. I just didn’t know too much about it, and I was like, ‘Well, I don’t really need it. I’m not that sexually active.’”*.

Mobile unit staff also recognized these perceptions:

I heard a bunch of times: ‘I don’t have sex enough,’ or, ‘I don’t have condomless sex enough to justify a daily pill.’ I think that’s a calculation for a lot of people― how much am I at risk, versus does that warrant taking a medication every day? And if my risky encounters are few and far between… I think those are the folks who are less likely to follow the process.

(Staff focus group participant)

Beyond an individual calculation of HIV risk in the context of deciding about PrEP, other contributing factors to perceived health and HIV risk emerged: the behavior, or perceived behavior, of others in the community and stigma related to sexuality and HIV.

### Health behaviors

We identified among the study’s participants a shared perception of MSM as particularly sexually active. While some simply relayed this stereotype, (“*the culture is so sexually active*,” noted a 30-year-old, Hispanic/Latino, previous PrEP user), others made the explicit connection between that perception and their own PrEP choices, justifying non-use of PrEP because their sexual activity was not as extensive as what they perceived for other MSM. For example, two different 23-year-old, White patients who had never used PrEP said, “*some gay people are addicted to sex and are having so many sexual partners. And I don’t feel like I fit into that mold. So, in my mind, I’m like, “Oh, I don’t need it.”* and “*there’s probably folks who need it more than me, to be honest. I’m not having a lot of sex.”* Another (33-year-old, Hispanic/Latino, never used PrEP) perceived the medication as being only for those who “*know they can’t sexually control themselves.”* The references to addiction and lack of control suggest that stigma about sexual behavior may be contributing to these men’s PrEP decisions.

Other comments reflected stigma about HIV impacting health behaviors and, in turn, the ability of MSM to accurately evaluate and perceive their health needs. A 33-year-old, Hispanic/Latino patient who has never used PrEP noted that “*it might be difficult when the little vans say, ‘HIV Tests,’ outside the club, because people can be judged for going into them. ‘Why are people going in there to get checked for HIV? Because maybe they have it.’ So, the community doesn’t like them, ‘I’m not going to have relations with him,’ because they’re afraid, so they apply a stigma, that stigma, that’s what we’re talking about*.” In addition, one 30-year-old, Hispanic/Latino patient who previously used PrEP said, “*people get afraid of the result, so they don’t get tested at all.”*

However, the knowledgeable staff and affirming atmosphere of the mobile unit helped change HIV risk perception, and potentially experiences of internalized stigma, for some participants. The 24-year-old above who did not start PrEP when their PCP suggested it did eventually begin the medication after having an STI exposure and being tested on the mobile unit, highlighting the potential of the unit to provide care for patients at “teachable moments”: “*At the time I had multiple sexual partners, so [the nurse practitioner] was just kind of explaining like, ‘Hey, it’s probably a good idea that you do this. It’s sort of preventative, and it’s a safe option.’”* Having now taken PrEP, their perspective has changed: “*even if you’re not that sexually active, if you have more than one partner, I think it’s really important.”*

## Discussion

Mobile units are attractive alternatives to clinic-based care, especially for people facing social or logistical barriers to care in traditional settings. In our study population of young, predominantly Hispanic/Latino MSM in a high-HIV-burden area in the USA, mobile PrEP and sexual health service provision were highly acceptable and feasible. Among those participating in this study, the positive view of the unit’s services was fostered by themes of improved access and community, but low self-perception of risk and stigma still existed as barriers to engagement.

We applied the Gelberg-Andersen Behavioral Model for Vulnerable Populations to our data as a framework to understand the impact of the mobile unit. The unit improved health service utilization in this vulnerable population because it provided accessible, MSM-oriented care specific to the needs of this community, in the context of a supportive and welcoming atmosphere. This approach acknowledges the operative predisposing factors which are intrinsically tied to health service utilization for this patient population. Sexual orientation, for example, was tied to experiences of stigma, an enabling/impeding factor which subsequently affected health behaviors and perceived need for care. Reducing stigma by leveraging other enabling factors, including peer support at the time of testing and identify-affirming care, led to improved health evaluation, service engagement, and satisfaction with care. In particular, our data suggest that careful attention to the creation of affirming care environments for MSM is a crucial aspect of service provision for this population.

Many of our study participants have intersectional identities which, within the model’s predisposing category, may increase their vulnerability and barriers to care. For example, young (13–34 years old), Black/African American, and Hispanic/Latino MSM face particularly high HIV incidence and yet low awareness and use of PrEP [9, 27–29]. In 2020, only 9% of Black individuals and 16% of Hispanic/Latino individuals who could benefit from PrEP received a prescription; the rate for young people aged 16–24 was also 16% [2]. The improvement and expansion of mobile units, including focusing on at-risk populations in future outreach efforts, may help close these gaps and facilitate more equitable PrEP coverage for those who need it. Further research focused on enabling and need factors specific to youth and people of color is necessary.

Furthermore, perceived HIV risk – which, in our study, was associated with stigma about sexual behavior and HIV – is an ongoing barrier to PrEP uptake and retention in care for MSM, even in the context of mobile units. Previous literature has shown that interest in taking PrEP is associated with self-perceived HIV risk among MSM [5]. Notably, low perception of need and limited PrEP use was present even in this study population of MSM who had engaged with sexual health services and been informed about PrEP. Many of our participants have never used PrEP, despite being offered it on the van and having positive views towards it. Clearly, access is not everything; efforts to impact HIV risk perception and reduce stigma are important for improving PrEP uptake among MSM. This data underscores the need to reframe PrEP as a health promotion, rather than risk reduction, tool.

Furthermore, strategies to promote continued engagement with care and retention in PrEP will be critical to attaining EHE goals. A recent study found that almost 20% of those prescribed PrEP never filled their prescription, a gap which persists even when the prescription was fully covered by insurance [30]. Implementing suggestions from our study participants, such as a consistent schedule and appointment capabilities, may improve the utility of the mobile unit for prolonged care engagement.

This study should be considered in light of its strengths and limitations. We sampled participants with a variety of perspectives and roles and included both English and Spanish speakers. However, our focus on a convenience sample of MSM in an urban USA setting with a high rate of insurance coverage may not be generalizable to other settings, and this work did not include other high-priority populations, including those under 18 years or transgender or gender diverse people. Non-response bias may be another limitation, as those who accessed services on the mobile unit and were willing to speak with us about PrEP and their experiences as MSM may be different from those who chose not to use the mobile unit or participate in our study.

Our study of urban MSM demonstrates acceptance of and enthusiasm for mobile sexual health services among patients and staff and provides concrete steps to improve these services in the future. This work suggests that mobile services are a novel and viable approach to address some of the most pressing barriers to care for MSM. These qualitative data can inform future research as well as further expansion and implementation of this model, including for emerging health services, such as injectable PrEP and mpox vaccination. If access and a community-oriented, identity-affirming environment are prioritized during implementation, mobile units represent an innovative approach for HIV prevention and health promotion.

## Electronic supplementary material

Below is the link to the electronic supplementary material.

Supplementary Material 1

## Data Availability

The datasets generated and analyzed during the current study are not publicly available to protect the confidentiality of our study participants. However, the dataset may be available from the corresponding author on reasonable request and contingent upon institutional review board approval for data sharing.
