# Supplementary material for: “That was our afterparty”: a qualitative study of mobile, venue-based PrEP for MSM
Source: BMC Health Serv Res. 2023 May 17;23:504. doi: 10.1186/s12913-023-09475-8 (PMC10191083; doi:10.1186/s12913-023-09475-8)
Supplement: Supplementary file 1 — Supplementary Material 1 [file 12913_2023_9475_MOESM1_ESM.docx]

**ADDITIONAL FILE 1**

**Consolidated criteria for reporting qualitative studies (COREQ): 32-item checklist**

| **Item** | **Guide questions/description** | **Reported on Page #** |
| --- | --- | --- |
| **Domain 1: Research team and reﬂexivity** | | |
| *Personal Characteristics* | | |
| Inter viewer/facilitator | Which author/s conducted the interview or focus group? | 5 |
| Credentials | What were the researcher’s credentials? | 1 (Affiliations), 5 |
| Occupation | What was their occupation at the time of the study? | 5 |
| Gender | Was the researcher male or female? | 5 |
| Experience and training | What experience or training did the researcher have? | 5 |
| *Relationship with participants* |  |  |
| Relationship established | Was a relationship established prior to study commencement? | 5 |
| Participant knowledge of the interviewer | What did the participants know about the researcher? e.g. personal goals, reasons for doing the research | 5 (Participants were briefed on the purpose of the study and prompted to ask any questions they had about the study or the interviewer.) |
| Interviewer characteristics | What characteristics were reported about the interviewer/facilitator? (e.g., bias, assumptions, reasons and interests in the research topic) | No |
| **Domain 2: study design** | | |
| *Theoretical framework* | | |
| Methodological orientation and Theory | What methodological orientation was stated to underpin the study? e.g. grounded theory, discourse analysis, ethnography, phenomenology, content analysis | 6-8 |
| *Participant selection* | | |
| Sampling | How were participants selected? e.g. purposive, convenience, consecutive, snowball | 5 |
| Method of approach | How were participants approached? e.g. face-to-face, telephone, mail, email | 5 |
| Sample size | How many participants were in the study? | 5, Figure 1 |
| Non-participation | How many people refused to participate or dropped out? Reasons? | Figure 1 |
| *Setting* | | |
| Setting of data collection | Where was the data collected? e.g. home, clinic, workplace | 5-6  Interviews were conducted over the phone while the participant was in a private setting (usually their home); focus groups were conducted through an audio-only Zoom call while staff were in clinic and patients were in a private setting. |
| Presence of non-participants | Was anyone else present besides the participants and researchers? | No |
| Description of sample | What are the important characteristics of the sample? e.g. demographic data, date | Table 2 |
| *Data collection* | | |
| Interview guide | Were questions, prompts, guides provided by the authors? Was it pilot tested? | Table 1 |
| Repeat interviews | Were repeat interviews carried out? If yes, how many? | No |
| Audio/visual recording | Did the research use audio or visual recording to collect the data? | 7 |
| Field notes | Were ﬁeld notes made during and/or after the interview or focus group? | No |
| Duration | What was the duration of the inter views or focus group? | 6 |
| Data saturation | Was data saturation discussed? | 8 |
| Transcripts returned | Were transcripts returned to participants for comment and/or correction? | No |
| **Domain 3: analysis and ﬁndings** | | |
| *Data analysis* | | |
| Number of data coders | How many data coders coded the data? | 7 |
| Description of the coding tree | Did authors provide a description of the coding tree? | No |
| Derivation of themes | Were themes identiﬁed in advance or derived from the data? | 7 |
| Software | What software, if applicable, was used to manage the data? | 7 |
| Participant checking | Did participants provide feedback on the ﬁndings? | No |
| *Reporting* | | |
| Quotations presented | Were participant quotations presented to illustrate the themes/ﬁndings? Was each quotation identiﬁed? e.g. participant number | Yes |
| Data and ﬁndings consistent | Was there consistency between the data presented and the ﬁndings? | Yes |
| Clarity of major themes | Were major themes clearly presented in the ﬁndings? | Yes, throughout |
| Clarity of minor themes | Is there a description of diverse cases or discussion of minor themes? | No |
